# Iron restriction induces preferential down-regulation of H_2_-consuming over H_2_-evolving reactions during fermentative growth of *Escherichia coli*

**DOI:** 10.1186/1471-2180-11-196

**Published:** 2011-08-31

**Authors:** Constanze Pinske, Gary Sawers

**Affiliations:** 1 Institute for Microbiology, Martin-Luther University Halle-Wittenberg, Kurt-Mothes-Str. 3, 06120 Halle (Saale), Germany

## Abstract

**Background:**

*Escherichia coli *synthesizes three anaerobically inducible [NiFe]-hydrogenases (Hyd). All three enzymes have a [NiFe]-cofactor in the large subunit and each enzyme also has an iron-sulfur-containing small subunit that is required for electron transfer. In order to synthesize functionally active Hyd enzymes iron must be supplied to the maturation pathways for both the large and small subunits. The focus of this study was the analysis of the iron uptake systems required for synthesis of active Hyd-1, Hyd-2 and Hyd-3 during fermentative growth.

**Results:**

A transposon-insertion mutant impaired in hydrogenase enzyme activity was isolated. The mutation was in the *feoB *gene encoding the ferrous iron transport system. The levels of both hydrogen-oxidizing enzymes Hyd-1 and Hyd-2 as determined by specific in-gel activity staining were reduced at least 10-fold in the mutant after anaerobic fermentative growth in minimal medium, while the hydrogen-evolving Hyd-3 activity was less severely affected. Supplementation of the growth medium with ferric iron, which is taken up by e.g. the siderophore enterobactin, resulted in phenotypic complementation of the *feoB *mutant. Growth in rich medium demonstrated that a mutant lacking both the ferrous iron transport system and enterobactin biosynthesis (*entC*) was devoid of Hyd-1 and Hyd-2 activity but retained some hydrogen-evolving Hyd-3 activity. Analysis of crude extracts derived from the *feoB entC *double null mutant revealed that the large subunits of the hydrogen-oxidizing enzymes Hyd-1 and Hyd-2 were absent. Analysis of *lacZ *fusions demonstrated, however, that expression of the *hya*, *hyb *and *hyc *operons was reduced only by maximally 50% in the mutants compared with the wild type.

**Conclusions:**

Our findings demonstrate that the ferrous iron transport system is the principal route of iron uptake for anaerobic hydrogenase biosynthesis, with a contribution from the ferric-enterobactin system. Hydrogen-oxidizing enzyme function was abolished in a *feoB entC *double mutant and this appears to be due to post-translational effects. The retention of residual hydrogen-evolving activity, even in the *feoB entC *double null mutant suggests that sufficient iron can be scavenged to synthesize this key fermentative enzyme complex in preference to the hydrogen-uptake enzymes.

## Background

[NiFe]-hydrogenases catalyze the reversible activation of molecular hydrogen [1]. The genome of *Escherichia coli *encodes four membrane-associated [NiFe]-hydrogenases, only three of which are synthesized under standard anaerobic growth conditions. Two of these enzymes, hydrogenase 1 (Hyd-1) and Hyd-2, oxidize hydrogen while the third, Hyd-3, is part of the hydrogen-evolving formate hydrogenlyase (FHL) complex [2], which disproportionates formic acid into CO_2 _and H_2 _and is an important means of preventing acidification of the cytoplasm during mixed-acid fermentation. While all three Hyd enzymes are synthesized during fermentation Hyd-3 appears to contribute the bulk (80-90%) of the measureable hydrogenase activity (measured as H_2_: benzyl viologen oxidoreductase activity) under these conditions, with Hyd-2 and Hyd-1 contributing the remainder [3]. Moreover, it has been recently demonstrated that Hyd-2 is functional in hydrogen oxidation at more reducing redox potentials while Hyd-1 is optimally active at more oxidizing potentials and is less oxygen-sensitive than Hyd-2 [4]. This presumably provides the bacterium with the capability of oxidizing hydrogen over a broad range of redox potentials.

The active site of the [NiFe]-hydrogenases comprises a Ni atom and a Fe atom to which the diatomic ligands CO and CN^- ^are attached [5]. The Hyp proteins synthesize this hetero-bimetallic centre and mutations in the genes encoding these Hyp maturases result in a hydrogenase-negative phenotype [2,5]. Iron is also required as a key component of the [Fe-S] clusters in the respective electron-transferring small subunits of the hydrogenases [5,6]. In addition, iron is required for the function of at least one of the Hyp maturases, HypD [7,8].

While the route of nickel transport for hydrogenase biosynthesis in *E. coli *has been well characterized [5,9], it has not been determined which of the characterized iron uptake systems is important for delivering iron to the hydrogenase maturation pathway. *E. coli *has a number of iron transport systems for the uptake of both ferric and ferrous iron [10]. Under anaerobic, reducing conditions Fe^2+ ^is the predominant form of iron and it is transported by the specific ferrous-iron FeoABC transport system [11,12]. Under oxidizing conditions, where the highly insoluble Fe^3+ ^is the form that is available, *E. coli *synthesizes Fe^3+^-specific siderophores to facilitate iron acquisition [13]. These Fe^3+^-siderophore complexes are transported into the cell by specific transport systems, e.g. Fe^3+^-citrate is transported by the Fec system, Fe^3+^-hydroxamate by the Fhu system and Fe^3+^-enterobactin by the Fep system. In this study we examined the biosynthesis and activities of the [NiFe]-hydrogenases during fermentative growth in null mutants lacking defined iron transport systems.

## Results

### A *feoB *mutant has reduced hydrogenase activity in both minimal and rich medium

All three [NiFe]-hydrogenases in *E. coli *catalyze the hydrogen-dependent reduction of the artificial redox dye benzyl viologen (BV) [3,14]. This activity can be visualized in colonies on agar plates after anaerobic fermentative growth. The colonies of wild type cells develop a dark violet colour in the presence of hydrogen and BV, while mutants unable to synthesize hydrogenase remain colourless [15]. Approximately 4000 kanamycin-resistant Tn*5*-insertion mutants were screened for an impaired ability to catalyze the hydrogen-dependent reduction of BV after anaerobic fermentative growth on M9 minimal medium plates with glucose as carbon source (see Methods for details). One of eight putative mutants isolated had a pale violet colony colour after BV-overlay in the presence of hydrogen; the characterization of the remaining seven putative mutants will be described elsewhere. Transduction of the mutation in the pale-violet mutant into a 'clean' MC4100 genetic background resulted in the mutant PM06, which retained the phenotype of the originally isolated mutant. Sequence analysis of the site of Tn*5 *insertion in the mutant revealed that it had inserted in the *feoB *gene, which encodes the GTPase component of the ferrous iron transporter [12].

The hydrogen-dependent reduction of BV was determined in extracts derived from MC4100 (wild type) and PM06 (*feoB*::Tn*5*) grown anaerobically in M9 minimal medium with glucose as carbon source and with different iron sources (Table 1). The wild type MC4100 grown without addition of iron compounds had a total hydrogenase activity of 2.0 U mg of protein^-1 ^(Table 1). Growth of MC4100 in the presence of iron citrate and potassium ferricyanide had essentially no effect on enzyme activity, while ferric chloride resulted in an 80% increase and ferric ammonium sulfate a 1.6-fold increase in total hydrogenase activity (Table 1). Growth of MC4100 in the presence of potassium ferrocyanide (Fe^2+^) resulted in extracts with a reduced but still significant hydrogen-oxidizing activity of 66% compared to the wild type grown without addition. It was noted that due to the poor growth of the strains in minimal medium in the presence of ferricyanide and ferrocyanide the hydrogenase enzyme activity was highly variable with high SD values. This phenomenon was reproducibly observed, despite attempts to harvest cells under strictly comparable conditions of growth and presumably reflects variability in the labile Hyd-3 activity (see below). Therefore, it must be stressed that only general trends in enzyme activity changes caused by these iron sources can be considered.

**Table 1:** Hydrogen-oxidizing enzyme activity of the *feoB*::Tn*5 *mutant PM06 grown in minimal medium with different iron sources

Strain and iron supplement^a^	Hydrogenase specific activity^b^ (μmol H_2 _oxidized min^-1 ^mg protein^-1^)	
	MC4100	PM06 (*feoB*::Tn*5*)
no iron addition	2.02 ± 0.64	0.49 ± 0.19
7.5 μM iron chloride (FeCl_3_)	3.63 ± 0.73	2.49 ± 0.64
15.3 μM hemin	1.72 ± 0.92	0.25 ± 0.18
10 μM potassium ferrocyanide (K_4_[Fe(CN)_6_]) (Fe^2+^)	1.34 ± 1.30	0.38 ± 0.33
10 μM potassium ferricyanide (K_3_[Fe(CN)_6_]) (Fe^3+^)	1.80 ± 2.82	0.93 ± 0.85
10 μM ferric ammonium sulfate (Fe(NH_4_)(SO_4_)_2_)	3.33 ± 2.53	2.02 ± 2.11
50 μM iron citrate (C_6_H_5_FeO_7_)	2.20 ± 0.70	3.47 ± 1.17
300 μM 2,2'-dipyridyl	< 0.01	< 0.01
300 μM 2,2'-dipyridyl and 200 μM FeCl_3_	0.04 ± 0.07	< 0.01
300 μM 2,2'-dipyridyl and 200 μM iron citrate	1.59 ± 1.16	0.04 ± 0.06

^a ^Cells were cultivated in M9 minimal medium including 0.8% (w/v) glucose. Iron sources were added at the given final concentrations.

^b ^The activities were determined for triplicate experiments.

Extracts of a *hypF *mutant, which cannot synthesize active hydrogenases [16], had essentially no hydrogenase enzyme activity and served as a negative control. Extracts of the *feoB*::Tn*5 *mutant PM06 grown in M9 medium in the absence of iron had a total hydrogenase activity that was 24% that of the wild type without addition of iron compounds (Table 1). Growth of PM06 in the presence of iron chloride or ferric ammonium sulfate restored hydrogenase activity to levels similar to wild type. The exception was potassium ferricyanide, which failed to restore hydrogenase enzyme activity to wild type levels; instead activity was approximately 50% of that measured in MC4100 grown without iron supplementation and only 50% of that measured after growth of the wild type with potassium ferricyanide (Table 1). In contrast, growth of PM06 in the presence of ferrocyanide did not restore hydrogenase activity. Addition of hemin as a source of oxidized iron also failed to restore hydrogenase activity to PM06, presumably because hemin cannot be taken up by *E. coli *and the oxidized iron is also tightly bound to the porphyrin. Taken together, these results are consistent with the ferrous iron transport system being an important route of iron uptake for hydrogenase biosynthesis in the wild type.

Addition of 2, 2'-dipyridyl to the growth medium resulted in total loss of hydrogenase activity of the wild type MC4100 and PM06 (Table 1). Supplementation of 200 μM iron chloride or iron citrate together with 300 μM dipyridyl showed that iron citrate restored 66% of the wild type activity while iron chloride failed to restore activity. None of these additions restored hydrogenase activity to PM06.

**Figure 1:**
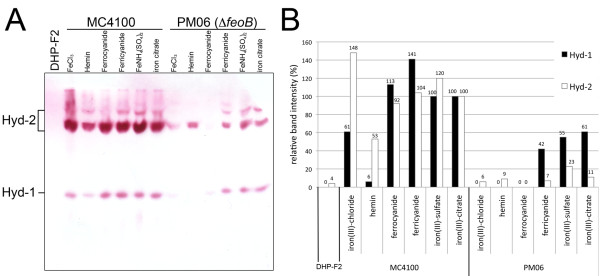
**Effect of different iron supplements on Hyd-1 and Hyd-2 activities in PM06 (*feoB*::Tn*5*) after growth in M9 minimal medium**. (A) Aliquots of crude extracts (25 μg) derived from DHP-F2 (negative control) the wild type (MC4100) and PM06 grown anaerobically in M9 minimal medium with glucose and the iron sources indicated were separated by non-denaturing PAGE (7.5% w/v polyacrylamide) and subsequently stained for hydrogenase enzyme activity (see Methods). The iron sources were the following: 7.5 μM FeCl_3_; 15.3 μM hemin; 50 μM iron citrate (C_6_H_5_FeO_7_) (Fe^3+^); 10 μM potassium ferrocyanide (K_4_[Fe(CN)_6_]) (Fe^2+^); 10 μM potassium ferricyanide (K_3_[Fe(CN)_6_]) (Fe^3+^); 10 μM Fe(NH_4_)(SO_4_)_2 _(Fe^3+^). **(B) **Densitometric quantification of the activity bands corresponding to Hyd-1 (black bars) and Hyd-2 (white bars) from the activity gel. Values were calculated as relative values compared to the intensity of the activity bands in the wild type (MC4100) grown with iron-citrate.

The activities of Hyd-1 and Hyd-2 can be visualized after non-denaturing PAGE followed by specific activity staining [14]; Hyd-3 is labile and cannot be visualized under these conditions. This method allows a specific analysis of the effect of mutations or medium supplements on Hyd-1 and Hyd-2 activity and it should be noted that this method is only semi-quantitative. Analysis of the extracts of MC4100 grown in minimal medium under all of the conditions tested identified both Hyd-1 and Hyd-2 as characteristic hydrogen-oxidizing activity-staining bands (Figure 1A). The relative intensity of the activity-staining bands was quantified by densitometric analysis (Figure 1B) as described in the Methods section. The intensity of the Hyd-1 and Hyd-2 activity-staining bands was similar when cells were grown fermentatively in the presence of iron citrate, ferric ammonium sulfate, ferricyanide or ferrocyanide. In cell-free extracts derived from PM06 grown with the three Fe^3+ ^sources ferricyanide, ferric ammonium sulfate and ferric citrate the Hyd-1 activity-staining profile was similar to that of the wild type, however, the intensity was reduced by approximately 50% (Figure 1). On the other hand, Hyd-2 attained a level that was only between 10 and 20% the intensity of the wild type grown with iron citrate, suggesting that the activity of this enzyme is less readily complemented by addition of oxidized iron. Somewhat surprising, however, was the observation that although some activity of Hyd-2 could be observed after growth of the mutant in the presence of FeCl_3_, Hyd-1 activity was strongly reduced (Figure 1). Total hydrogenase enzyme activity measured in these extracts of PM06 was nevertheless near wild type (Table 1). It must be noted, however, that under these growth conditions the contributions of Hyd-1 and Hyd-2 to the total activity are low (around 1% for Hyd-1 and 5-10% for Hyd-2), as can be deduced from a strain lacking Hyd-3 (CP971) that retained 4% of the wild type activity with iron chloride [3,17]. This means that although Hyd-1 or Hyd-2 activities could barely be observed by in-gel staining, the increase in total hydrogenase activity by addition of FeCl_3 _was due to Hyd-3 activity.

An extract derived from PM06 grown in the presence of ferrocyanide showed essentially no detectable activity due to either Hyd-1 or Hyd-2 (Figure 1), indicating that probably the level of iron in the mutant was insufficient to allow synthesis of wild type levels of these enzymes. This correlated with the low total hydrogenase activity measured in extracts of PM06 after fermentative growth with ferrocyanide, and indicates that the residual activity was due to Hyd-3 (Table 1). After growth of PM06 in the presence of hemin no Hyd-1 activity was detected in the gel (Figure 1), and only a very low Hyd-2 activity was detected. Total hydrogenase activity was only 10% of the total compared to wild type without addition of iron compounds, indicating that Hyd-3 activity was not recovered in PM06 by addition of hemin to the growth medium.

The effect of the *feoB *mutation on hydrogenase enzyme activity could also be observed after growth in rich medium, whereby the hydrogenase enzyme activity of the *feoB *mutant PM06 was reduced by a little over 50% compared with the activity of MC4100 (Table 2).

**Table 2:** Hydrogen-oxidizing enzyme activity of the complemented PM06 (*feoB*::Tn*5*) mutant

Strain^a ^and genotype	Hydrogenase specific activity^b^ (μmol H_2 _oxidized min^-1 ^mg protein^-1^)
MC4100	2.96 (± 0.31)
DHP-F2 (*hypF*)	< 0.01
PM06 (*feoB*::Tn*5*)	1.28 (± 0.50)
PM06 pECD1079 (*feoB*^+^)	0.44 (± 0.13)
PM06 pFEO (*feoABC*^+^)	3.4 (± 1.30)

^a ^Cell extracts were prepared from cells grown anaerobically in TGYEP plus formate.

^b ^The mean and standard deviation of at least three independent experiments are shown.

In an attempt to complement the *feoB *mutation, initially the *feoB *gene was re-introduced into PM06 by transformation of plasmid pECD1079 (*feoB*^+^). The plasmid failed to restore hydrogenase enzyme activity to the levels determined for the wild type; surprisingly, the presence of the plasmid reduced overall hydrogenase activity to only about 15% that of the wild type (Table 2). Western blot analysis of the Strep-tagged FeoB derivative encoded on pECD1079 confirmed that the protein was synthesized but that the level of synthesis was higher in aerobically grown cells compared with anaerobically grown cells (Additional file 1). The reason for the reduction in hydrogenase activity caused by over-produced Strep-tagged FeoB is unclear.

Introduction of the complete *feoABC *operon on the plasmid restored hydrogenase activity in PM06 to wild type levels (Table 2). This latter result suggests that the transposon insertion in the *feoB *gene caused a polar effect on the downstream *feoC *gene and only the presence of the complete operon on a plasmid could complement the mutation.

### Combined knock-out of ferrous and ferric iron transport systems abolishes hydrogen-oxidizing activities

Single null mutations that prevented biosynthesis of ferric-enterobactin (strain CP416 Δ*entC*) or the uptake system for ferric-citrate (strain CP422, Δ*fecA-E*) essentially had little to no effect on total hydrogenase activity (Table 3). Introducing a mutation in the *fhuA *or *fhuE *genes also had no effect on total hydrogenase activity (data not shown). Combining the *entC *and *fecA-E *mutations (strain CP415) reduced hydrogenase activity by approximately 60% compared to the wild type. Introducing the *feoB*::Tn*5 *mutation into this strain to deliver CP413 (*entC fecA-E feoB*::Tn*5*) reduced total hydrogenase activity even further such that only approximately 7% of the wild type level could be detected.

**Table 3:** Hydrogen-oxidizing enzyme activity in various transport mutants

Strain^a ^and genotype	Hydrogenase Specific activity^b^ (μmol H_2 _oxidized min^-1 ^mg protein^-1^)
MC4100	2.70 ± 0.8
DHP-F2 (*hypF*)	0.02 ± 0.01
PM06 (*feoB*)	1.24 ± 1.0
CP422 (*fecA-E*)	2.54 ± 1.6
CP416 (*entC*)	2.05 ± 0.5
CP411 (*entC feoB*)	0.58 ± 0.4
CP415 (*fecA-E entC*)	1.11 ± 0.4
CP413 (*entC feoB fecA-E*)	0.19 ± 0.16

^a ^Cell extracts were prepared from cells grown anaerobically in TGYEP plus 15 mM formate.

^b ^The mean and standard deviation of at least three independent experiments are shown.

**Figure 2:**
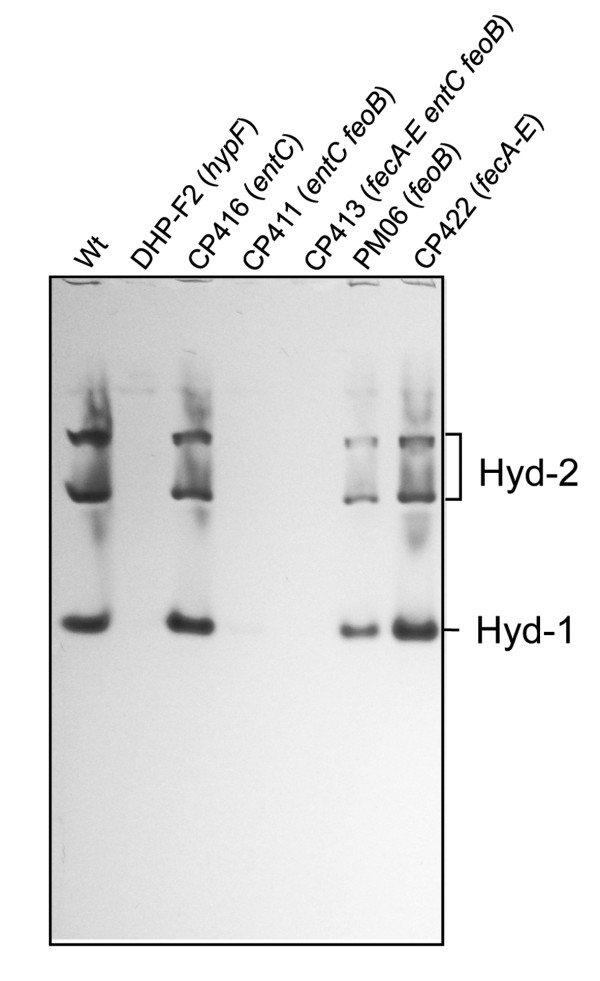
**Hyd-1 and Hyd-2 activities in iron transport mutants after growth in rich medium**. Aliquots of crude extracts (25 μg of protein) derived from each of the mutants grown by fermentation in TGYEP medium, pH 6.5, were separated by non-denaturing PAGE (7.5% w/v polyacrylamide) and stained for hydrogenase activity as described in the Methods section. The stained bands corresponding to active Hyd-1 and Hyd-2 are indicated. The name of the mutants and the corresponding mutated genes are indicated above each lane.

Analysis of cell-free extracts derived from these strains grown fermentatively in rich medium by non-denaturing PAGE, with subsequent staining for activity of Hyd-1 and Hyd-2, revealed that, as anticipated, the extracts of CP416 (*entC*) and CP422 (*fecA-E*) showed essentially wild-type Hyd-1 and Hyd-2 activity profiles (Figure 2). However, an extract from PM06 (*feoB*::Tn*5*) showed clearly reduced intensity bands for both enzymes, which is in accord with the results after growth in minimal medium (see Figure 1). Extracts from CP411 (*entC feoB*::Tn*5*) or CP413 (*entC fecA-E feoB*::Tn*5*) grown fermentatively in rich medium had neither Hyd-1 nor Hyd-2 enzyme activities. This result indicates that the residual hydrogenase enzyme activity in CP413 must result from Hyd-3 (compare Table 3). To test this, we determined the FHL enzyme activity present in whole cells of the various mutants (Table 4) and could demonstrate that while cells of CP411 (*entC feoB*::Tn*5*) had an FHL activity of approximately 50% of the wild-type, strain CP413 (*entC fecA-E feoB*::Tn*5*) still retained 30% of the wild-type FHL activity, confirming that the residual hydrogenase activity in extracts of CP413 was indeed due to Hyd-3.

**Table 4:** Formate hydrogenlyase activity of the transport mutants

Strain^a^	Specific hydrogen evolving activity (mU mg protein^-1^)^b^
MC4100	30 ± 7
DHP-F2 (*hypF*)	< 1
CP416 (*entC*)	20 ± 5
PM06 (*feoB*)	15 ± 3
CP411 (*entC feoB*)	15 ± 6
CP413 (*entC feoB fecA-E*)	9 ± 1

^a ^Cells were grown anaerobically in TGYEP.

^b ^The mean and standard deviation of at least three independent experiments are shown.

### Hyd-1 and Hyd-2 large subunits are absent in CP413 (*entC feoB*:: Tn*5*)

**Figure 3:**
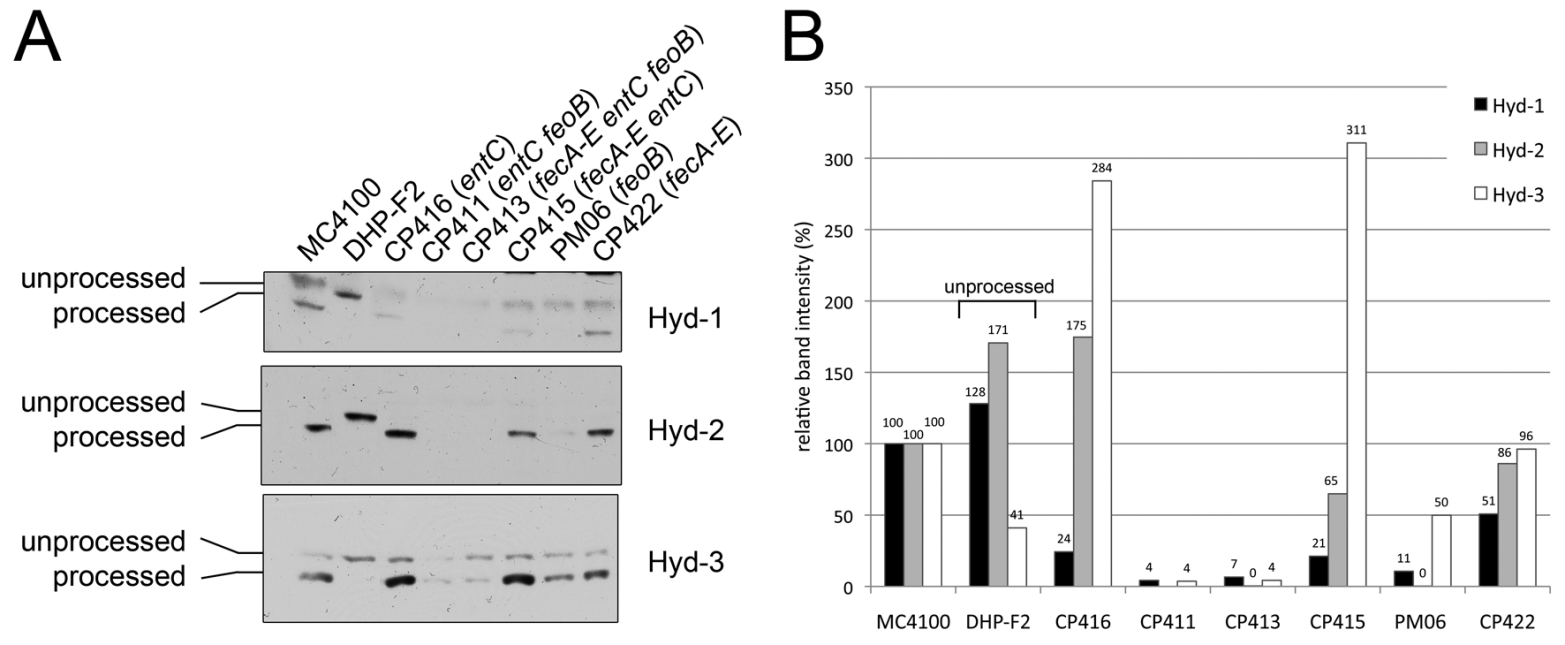
**Analysis of hydrogenase large subunit processing**. **(A) **The three panels show portions of Western blots in which the large subunits of Hyd-1, Hyd-2 and Hyd-3 (HycE) are shown. The positions of the unprocessed and processed forms of the polypeptides are indicated on the left of the Figure. Crude extracts (25 μg of protein) derived from cells grown anaerobically in TGYEP plus formate were separated in 10% (w/v) SDS-PAGE and incubated with antibodies specific for the respective enzymes. **(B) **Densitometric quantification of the processed protein bands (and for the unprocessed band from DHP-F2) corresponding to Hyd-1 (black bars), Hyd-2 (gray bars) and Hyd-3 (white bars) from the western blot. Values were calculated as relative intensities compared to the intensity of the wild type MC4100.

In order to determine whether the lack of Hyd-1 and Hyd-2 activity in the mutants devoid of ferrous and ferric uptake was due to lack of processing of the large subunits because of iron-limitation, the precursor and processed forms of the large subunits of Hyd-1, Hyd-2 and Hyd-3 in cell-free extracts derived from the iron transport mutants after growth in rich medium were analysed by Western blotting with enzyme-specific antisera. Extracts derived from MC4100 (wild type) revealed mainly the processed form of the catalytic subunit of all three enzymes (Figure 3A), which is indicative of successful insertion of the [NiFe]-cofactor [5]. In contrast, a mutant unable to synthesize the HypF protein (DHP-F2) is unable to generate the diatomic CN^- ^ligands and consequently fails to insert the cofactor. Extracts from a *hypF *mutant therefore only showed the unprocessed form of each catalytic subunit (Figure 3A), which indicates that the large subunit lacks a cofactor [5]. Extracts derived from CP416 (*entC*) and CP422 (*fecA-E*) both showed levels of processed large subunits for Hyd-1, Hyd-2 and Hyd-3 similar to those seen for the wild-type MC4100 (Figure 3A). Densitometric analysis of the levels of these processed polypeptides in the autoradiogram shown in Figure 3A, however, revealed that in extracts of CP416 and CP422 Hyd-1 large subunit levels were only 20% and 50%, respectively, of that observed in the wild type, while in extracts of CP416 the level of Hyd-3 large subunit HycE was almost 3-fold increased compared with the level in the wild type (Figure 3B). Extracts derived from the *fecA-E entC *double null mutant CP415 showed the similar increased level of Hyd-3 large subunit and decreased level of Hyd-1 large subunit as was observed with CP416; however, the difference was that Hyd-2 levels were decreased by approximately 40% compared with the wild type. These results suggest that under mild iron-limiting conditions, intracellular iron is preferentially used for hydrogen-evolving function. The *feoB *mutant PM06 showed strongly reduced levels of processed Hyd-1 large subunit and barely detectable levels of Hyd-2 processed large subunit; the amount of processed Hyd-3 large subunit was approximately 50% that of the wild-type. Cell-free extracts of CP411 (*entC feoB*::Tn*5*) and CP413 (*entC fecA-E feoB*::Tn*5*), on the other hand, essentially completely lacked either the unprocessed or processed forms of the large subunits of Hyd-1 or Hyd-2, which correlates with the lack of Hyd-1 and Hyd-2 enzyme activity observed in Figure 2. Both the processed and unprocessed forms of the Hyd-3 large subunit HycE were observed in extracts from both strains but at significantly reduced levels, which is in accord with the observed FHL activity measured in the strains (see Table 4). Although the level of processed HycE in both strains was significantly reduced compared with the wild-type, the amounts of full-length, unprocessed polypeptide were similar to those seen in the wild-type (Figure 3A), suggesting that there was a limitation in processing capacity in the mutants.

### Expression of the *hya*, *hyb *and *hyc *operons is only marginally reduced in the iron-transport mutants

The *hya*, *hyb *and *hyc *operons encode Hyd-1, Hyd-2 and Hyd-3, respectively [2,18,19]. To determine whether expression of these operons was affected in the different iron-transport-defective mutants, we constructed *lacZ *translational fusions to the first gene of each operon, which encode the respective small subunits of the enzymes Hyd-1 and Hyd-2, while the *hycA *gene encodes a transcriptional regulator (see Methods). After transfer to the lambda phage λRS45 [20], the *hyaA'-'lacZ*, *hybO'-'lacZ *and *hycA*'-'*lacZ *fusions were introduced in single copy onto the chromosome of the respective mutants. To demonstrate that the fusions were functional we analyzed expression levels after growth under both aerobic and anaerobic conditions. Expression of *hyaA*'-'*lacZ *was strongly reduced when wild type cells were grown aerobically, while expression was up-regulated approximately 70-80 fold during fermentative growth (Table 5). The *hybO*'-'*lacZ *expression was shown to be approximately 5 fold higher in anaerobically grown compared with aerobically grown cells. Expression of *hycA*'-'*lacZ *was up-regulated 3 fold in the presence of formate. All fusions showed near background β-galactosidase enzyme activity when cells were grown aerobically [21,22].

**Table 5:** Influence of iron transport mutations on expression of *hyaA*, *hybO *and *hycA lacZ *fusions

	β-Galactosidase specific activity in Miller Units (± standard deviation)		
Strain/genotype^a^	Φ(*hyaA*'-'*lacZ*)	Φ(*hybO*'-'*lacZ*)	Φ(*hycA*'-'*lacZ*)
MC4100 (wild type)	818 ± 232	52 ± 46	44 ± 9
MC4100 aerobically	12 ± 3	12 ± 3	13 ± 2
MC4100 + 15 mM formate	770 ± 535	87 ± 30	126 ± 57
DHP-F2 (Δ*hypF*)	620 ± 221	60 ± 27	53 ± 22
Δ*fecA-E*	633 ± 252	52 ± 17	41 ± 11
Δ*feoB*	355 ± 96	36 ± 7	65 ± 40
Δ*entC*	410 ± 110	40 ± 15	33 ± 20
Δ*fecA-E feoB*	491 ± 139	43 ± 11	28 ± 13
Δ*entC fecA-E feoB*	371 ± 94	45 ± 11	35 ± 24
Δ*entC feoB*	574 ± 155	45 ± 21	49 ± 32
Δ*entC fecA-E*	340 ± 211	47 ± 12	57 ± 19

^a ^In the interest of clarity only the genotype of the strains is given. The strains used can be found in Table 6. Growth was performed under fermentative conditions in TGYEP, unless indicated otherwise.

n. d.-not determined

The results in Table 5 show that in *entC *or *feoB *mutants, expression of *hyaA *was reduced by approximately 50% compared with the wild type MC4100. Expression of *hybO *attained levels that were only approximately 10% those of *hyaA *(Table 5), consistent with transcriptional regulation data for these operons reported earlier [21]. The expression of the *hybO'-'lacZ *fusion was reduced by approximately 40% in a *feoB *mutant background and by 35% in an *entC *mutant compared with the level of expression measured in the wild type (Table 5). Expression of the *hyc *operon remained comparatively constant among the strains, but was reduced by maximally 40% in a *fecA-E feoB *double mutant. A slight increase in *hyc *expression in the *feoB *single mutant was observed; however, it should be noted that expression levels were variable in the mutant backgrounds. Addition of dipyridyl to the growth medium had no effect on *hyc *expression (data not shown).

## Discussion

In a previous study [23] it was shown that hydrogen metabolism of *E. coli *was significantly affected by introduction of a *fur *mutation. Fur is a global regulator controlling iron homeostasis [24,25]. Differential effects on hydrogen-oxidizing hydrogenase activity compared with hydrogen-evolving enzyme function were observed previously in the *fur *mutant [23]. The *fur *mutation, which has both negative and positive effects on gene expression of iron metabolism including depression of iron uptake systems, caused a strong reduction in FHL activity, suggesting Fur is required for FHL synthesis. In the current study we could show in an otherwise Fur^+ ^background that causing iron limitation by removing key iron uptake systems also resulted in differential effects on hydrogen uptake and hydrogen evolution: hydrogen-oxidizing hydrogenase function was compromised first while hydrogen-evolving hydrogenase activity was partially retained. During a search for genes affecting hydrogenase biosynthesis or activity, a mutant with a transposon insertion in *feoB *encoding the GTPase component of the postulated ferrous iron transport system [12] was isolated. The alteration in hydrogen metabolism caused by the mutation could not be phenotypically complemented by ferrous iron but could be complemented by supplementing the growth medium with oxidized iron. This result supports the important role of the Feo system in transport of iron under reducing conditions. Although this finding was perhaps not surprising considering that the hydrogenases are synthesized under anaerobic fermentative conditions when Fe^2+ ^ions are available and the Feo transport system is active [10-12], it was nevertheless important to demonstrate the involvement and importance of this route of iron acquisition for enzymes that have a high demand for iron atoms.

Combining the *feoB *mutation with a mutation in *entC*, which impairs biosynthesis of the siderophore enterobactin, abrogated Hyd-1 and Hyd-2 activities. Notably, however, significant Hyd-3, and consequently FHL, activity was retained in the double null mutant, suggesting that when iron is limited during fermentative growth the synthesis of the hydrogen-evolving Hyd-3 takes precedence over the two hydrogen-oxidizing enzymes Hyd-1 and Hyd-2. The fact that Hyd-2 is maximally active under more reducing conditions, while Hyd-1 is an oxygen-tolerant enzyme and is active at more positive redox potentials [4], did not influence this preference. Even when a further mutation preventing synthesis of the iron-citrate transport system was introduced, residual Hyd-3 and FHL activities were retained. Indeed, previous studies demonstrated that only when *zupT *and *mntH *mutations were also introduced into this background was FHL activity abolished [23]. This suggests that the FHL system can scavenge residual iron entering the cell through unspecific transport systems, but that these levels of iron either are insufficient for synthesis of Hyd-1 and Hyd-2 or that the iron is directed preferentially to Hyd-3 biosynthesis. Further studies will be required to elucidate which of these possibilities is correct.

A somewhat unexpected result of this study was the finding that under iron limitation no unprocessed species of the Hyd-1 or Hyd-2 large subunits were present and only very low amounts of the processed proteins were observed. This was unexpected because in *hyp *mutants, where active site biosynthesis cannot be completed [5], significant levels of the unprocessed form of the large subunit are always detected (for example see extracts of DHP-F2 in Figure 3). The fact that expression of translational *lacZ *fusions of the *hya *and *hyb *structural gene operons was largely unaffected by the deficiency in iron transport suggests that a different level of regulation in response to iron availability exists. This regulation might possibly be post-translational, for example through altered protein turnover due to insufficient iron.

## Conclusions

Mutants unable to acquire iron through the ferrous iron transport and siderophore-based uptake systems lacked the hydrogen-oxidizing enzymes Hyd-1 and Hyd-2 under anaerobic fermentative conditions. Iron limitation did not affect transcription of the *hya*, *hyb *or *hyc *operons. The Hyd-3 component of the FHL complex was less severely affected by defects in these iron uptake systems, indicating that a greater degree of redundancy in iron acquisition for this enzyme exists. Thus, when iron becomes limiting during fermentative growth synthesis of active Hyd-3 has priority over that of the hydrogen-oxidizing enzymes Hyd-1 and Hyd-2. This probably reflects a physiological requirement to maintain an active FHL complex to offset acidification of the cytoplasm caused by formate accumulation via disproportionation of the metabolite into the freely diffusible gaseous products CO_2 _and H_2_.

## Methods

### Strains, plasmids and growth conditions

All bacterial strains, plasmids and phages used in this study are listed in Table 6.

**Table 6:** Strains and plasmids used in this study

Strains/plasmids	Genotype	Reference
MC4100	F^- ^*araD139 *Δ(*argF-lac*)*U169 ptsF25 deoC1 relA1 flbB5301 rspL150 *^-^	[37]
DHP-F2	MC4100 Δ*hypF *59-629AA	[16]
XL1-Blue	*recA*1 *endA*1 *gyrA*96 *thi-*1 *hsdR*17 *supE*44 *relA*1 *lac *[F' *proAB lacIqZ*ΔM15 Tn*10 *(Tet^R^)]	Stratagene
PM06	Like MC4100 but *feoB*::Tn*5*	This study
PX06	Like XL1-Blue but *feoB*::Tn*5*	This study
CP411	Like MC4100 but Δ*entC*::*cat feoB*::Tn*5*	This study
CP413	Like MC4100 but Δ*fecA-E *Δ*entC*::*cat feoB*::Tn*5*	This study
CP415	Like MC4100 but Δ*fecA-E *Δ*entC*::*cat*	This study
CP416^a^	Like MC4100 but Δ*entC*::*cat*	This study
CP422	Like MC4100 but Δ*fecA-E *introduced from GG7	This study
GG7	W3110 Δ*fecA-E*::*kan*	G. Grass
CP971	MC4100 Δ*hycAI*::*kan*	[38]
CP612	Like MC4100 but Φ(*hyaA*'-'*lacZ*)	This study
CP775	Like MC4100 but Φ(*hybO*'-'*lacZ*)	This study
CP951	Like MC4100 but Φ(*hycA*'-'*lacZ*)	This study
CP1069	Like MC4100 but Δ*hypF *Φ(*hyaA*'-'*lacZ*)	This study
CP1084	Like MC4100 but Δ*hypF *Φ(*hybO*'-'*lacZ*)	This study
CP1149	Like MC4100 but Δ*hypF *Φ(*hycA*'-'*lacZ*)	This study
CP1073	Like MC4100 but Δ*fecA-E *Φ(*hyaA*'-'*lacZ*)	This study
CP1088	Like MC4100 but Δ*fecA-E *Φ(*hybO*'-'*lacZ*)	This study
CP1150	Like MC4100 but Δ*fecA-E *Φ(*hycA*'-'*lacZ*)	This study
CP1075	Like MC4100 but Δ*feoB*^b ^Φ(*hyaA*'-'*lacZ*)	This study
CP1090	Like MC4100 but Δ*feoB*^b ^Φ(*hybO*'-'*lacZ*)	This study
CP1151	Like MC4100 but Δ*feoB*^b ^Φ(*hycA*'-'*lacZ*)	This study
CP1071	Like MC4100 but Δ*entC *Φ(*hyaA*'-'*lacZ*)	This study
CP1086	Like MC4100 but Δ*entC *Φ(*hybO*'-'*lacZ*)	This study
CP1152	Like MC4100 but Δ*entC *Φ(*hycA*'-'*lacZ*)	This study
CP1079	Like MC4100 but Δ*fecA-E feoB*^b ^Φ(*hyaA*'-'*lacZ*)	This study
CP1094	Like MC4100 but Δ*fecA-E feoB*^b ^Φ(*hybO*'-'*lacZ*)	This study
CP1153	Like MC4100 but Δ*fecA-E feoB*^b ^Φ(*hycA*'-'*lacZ*)	This study
CP1081	Like MC4100 but Δ*entC feoB*^b ^Φ(*hyaA*'-'*lacZ*)	This study
CP1096	Like MC4100 but Δ*entC feoB*^b ^Φ(*hybO*'-'*lacZ*)	This study
CP1154	Like MC4100 but Δ*entC feoB*^b ^Φ(*hycA*'-'*lacZ*)	This study
CP1077	Like MC4100 but Δ*entC fecA-E *Φ(*hyaA*'-'*lacZ*)	This study
CP1092	Like MC4100 but Δ*entC fecA-E *Φ(*hybO*'-'*lacZ*)	This study
CP1155	Like MC4100 but Δ*entC fecA-E *Φ(*hycA*'-'*lacZ*)	This study
CP1083	Like MC4100 but Δ*entC fecA-E feoB*^b ^Φ(*hyaA*'-'*lacZ*)	This study
CP1098	Like MC4100 but Δ*entC fecA-E feoB*^b ^Φ(*hybO*'-'*lacZ*)	This study
CP1163	Like MC4100 but Δ*entC fecA-E feoB*^b ^Φ(*hycA*'-'*lacZ*)	This study
Plasmids		
pFEO	*feoABC*^+ ^from *E. coli *in pASK-IBA7	[39]
pECD 1079	*feoB*^+ ^from *E. coli *in pASK-IBA7	N. Taudte and G. Grass
pRS552	Km^R ^Ap^R ^*lacZ*^+ ^*lacY*^+ ^*lacA*^+^	[20]
phyaA552	like pRS552 but containing Φ(*hyaA*'-'*lacZ*)	This study
phybO552	like pRS552 but containing Φ(*hybO*'-'*lacZ*)	This study
pTL101	like pRS552 but containing Φ(*hycA*'-'*lacZ*), cloned from PstI within *hycA *to AvaII within *hycA*	[28]

^a ^P1 lysate from Δ*entC*::*cat *was obtained from G. Grass and N. Taudte

^b ^Similar to PM06, these strains were constructed using P1*kc *lysate grown on the Δ*feoB*::*kan *knockout mutant JW3372 from the Keio collection [40] with subsequent elimination of the kanamycin resistance cassette [41].

For the purposes of chromosomal and plasmid DNA isolation, *E. coli *was grown aerobically in Erlenmeyer flasks filled to maximally 10% of their volume with LB medium on a rotary shaker (250 rpm) and incubated at 37°C. Anaerobic growths were performed at 37°C in sealed bottles filled with anaerobic medium and under a nitrogen gas atmosphere. Cultures for determination of hydrogenase processing or for enzyme activity measurements were grown either in buffered TGYEP medium (1% w/v tryptone, 0.8% w/v glucose, 0.5% w/v yeast extract, 0.1 M potassium phosphate buffer) pH 6,5 [15] supplemented with 15 mM formate or in M9 minimal medium [26] containing 0.8% (w/v) glucose as carbon source, all standard amino acids at a final concentration of 0,04 mg/ml and 0.3 μM thiamine. When used for growth and screening for hydrogen metabolism mutants M9-glucose was supplemented with 0.29 mM citrulline, 0.89 mM uracil and was solidified with 1.5% (w/v) agar. All media were supplemented with 0.1% (v/v) SLA trace element solution [27] except when different iron sources were tested in which case FeCl_3 _was omitted from SLA and was replaced by the appropriate iron source at the concentration indicated. Dipyridyl was added at a final concentration of 300 μM. All growth media included 0.1 μM NiCl_2_. The antibiotics kanamycin, ampicillin, and chloramphenicol, when required, were added to the medium at the final concentrations of 50, 100, and 12.5 μg per ml, respectively. When indicated anhydrotetracycline (AHT) was added at the final concentration of 0.2 μg per ml.

### Construction of *hyaA'-'lacZ*, *hybO'-'lacZ *and *hycA*'-'*lacZ *translational fusions

The translational fusions to *hyaA *and *hybO *were constructed by amplifying the respective promotor regions and the nucleotides coding for the first 14 or 13 amino acids, respectively, by PCR using Phusion DNA polymerase (Finnzymes, Germany) and the oligonucleotides hya_regulat_up 5'-GCG GGA TCC GCG CAG AGA TTC GAA CTC TG-3', hya_regulat_down 5'-GCG GGA TCC TGA CGC CGC ATG GCC TGG TA-3', hybO_-217 5'-CTC GGA TCC TAT GGC CGG TTA TCG CCT C-3' and hybO_+38 5'-CTC GGA TCC ATG CCG TGA GAA TGG ATG A-3'. The resulting respective 565 bp and 274 bp fragments were digested with BamHI and ligated into pRS552 [20], which had been digested with BamHI and dephosphorylated with shrimp alkaline phosphatase (Roche, Germany). This procedure delivered plasmids phyaA552 and phybO552, respectively. The DNA sequence was verified by sequencing (Seqlab, Germany) and the insert transferred to λRS45 [20]. In a similar manner the *hycA*'-'*lacZ *fusion was constructed using plasmid pTL101 [28]. The resulting Φ(*hyaA*'-'*lacZ*), Φ(*hybO*'-'*lacZ*) and Φ(*hycA*'-'*lacZ*) protein fusions were introduced in single copy into the lambda attachment site of the respective mutants as indicated in Table 6.

### Isolation of mutants and identification of the transposon insertion site

XL1-Blue (Stratagene) was mutagenized using the < R6Kγ*ori*/KAN-2 > transposome (Epicentre Biotechnologies; [29]) according to the manufacturer's instructions. Subsequent to mutagenesis, cells were plated on M9-glucose minimal medium including the supplements described above and mutants containing transposon-insertions in the chromosome were resistant to kanamycin. Plates were incubated for 2 days at 37°C under a H_2_/CO_2 _(90%/10%) atmosphere (gas-generating kit, Oxoid) and kanamycin-resistant colonies were analysed via a soft-agar overlay technique with benzyl viologen (BV) at a final concentration of 0.5 mM and in a hydrogen atmosphere as described [15]. Colonies with a wild type hydrogenase phenotype developed a dark violet colour while hydrogenase-negative mutants remained creamy white. After purification of putative hydrogenase-negative colonies on LB agar the mutation was transduced into MC4100 using P1*kc *according to Miller [30] and the phenotype verified.

In order to determine the transposon insertion site, chromosomal DNA was isolated from the mutants [26], digested with KpnI, EcoRI or BamHI and religated. The ligation mixture was PCR amplified using primers KAN-2 FP-1 5'-ACC TAC AAC AAA GCT CTC ATC AAC C-3' and R6Kan-2 RP-1 5'-CTA CCC TGT GGA ACA CCT ACA-3' and the PCR product sequenced to determine the precise site of insertion.

### Preparation of cell extracts and determination of enzyme activity

Anaerobic cultures were harvested at an OD_600 nm _of approximately 0.8. Cells from cultures were harvested by centrifugation at 4,000 × g for 10 min at 4°C, resuspended in 2-3 ml of 50 mM MOPS pH 7.0 buffer and lysed on ice by sonication (30 W power for 5 minutes with 0.5 sec pulses). Unbroken cells and cell debris were removed by centrifugation for 15 min at 10, 000 × g at 4°C and the supernatant was used as the crude cell extract. Protein concentration of crude extracts was determined [31] with bovine serum albumin as standard. Hydrogenase activity was measured according to [14] except that the buffer used was 50 mM MOPS, pH 7.0. The wavelength used was 578 nm and an E_M _value of 8,600 M^-1 ^cm^-1 ^was assumed for reduced benzyl viologen. One unit of activity corresponded to the reduction of 1 μmol of hydrogen per min. Formate hydrogenlyase (FHL) activity was measured according to [23] using gas chromatography.

Beta-galactosidase assay was performed in microtiter plates according to [32] using a BioRad microplate reader Model 3550 (BioRad, Munich).

### Polyacrylamide gel electrophoresis and immunoblotting

Aliquots of 50 μg of protein from crude cell extracts were separated on 10% (w/v) SDS-polyacrylamide gel electrophoresis (PAGE) [33] and transferred to nitrocellulose membranes as described [34]. Membrane samples were treated with 2× SDS sample buffer [35] containing 10 mM DTT and incubated at room temperature for 60 min prior to loading onto the gel. Antibodies raised against Hyd-1 (1:10000), HycE (1:3000), Hyd-2 (1:20000; a kind gift from F. Sargent) or Strep-Tactin conjugated to horseradish peroxidase (IBA, Germany) were used. Secondary antibody conjugated to horseradish peroxidase was obtained from Bio-Rad. Visualisation was done by the enhanced chemiluminescent reaction (Stratagene).

Non-denaturating PAGE was performed using 7.5% (w/v) polyacrylamide gels pH 8.5 and included 0.1% (w/v) Triton-X100 in the gels [14]. Samples (25 μg of protein) were incubated with 5% (v/v) Triton X-100 prior to application to the gels.

Where indicated, the relative intensity of hydrogenase staining and protein amount from immunoblots was quantified using ImageJ from the National Institutes of Health [36].

Hydrogenase activity-staining was done as described in [14] except that the buffer used was 50 mM MOPS pH 7.0.

## Competing interests

The authors declare that they have no competing interests.

## Authors' contributions

CP carried out the experimental studies and helped draft the manuscript. GS conceived and coordinated the study and drafted the manuscript. Both authors read and approved the manuscript.

## Supplementary Material

Additional file 1 **Plasmid-encoded FeoB synthesis in MC4100 and PM06 (*feoB*::Tn*5*)**. Extracts (25 μg protein in membrane sample buffer) from MC4100 and PM06, transformed with pECD1079 bearing *feoB *and pFEO bearing the whole *feo *operon, both cloned behind a tetracycline promotor and encoding an *N*-terminal *StrepII*-tag on FeoB encoded on pECD1079 were separated by SDS-PAGE (10% w/v polyacrylamide) and after transfer to nitrocellulose detected by incubation with Strep-tactin conjugated to horseradish peroxidase. Strains were grown either with or without aeration in TGYEP, pH 6.5 and gene expression was induced with 0.2 μg ml^-1 ^AHT (anhydrotetracycline) as indicated. Biotin carboxyl carrier protein (BCCP) served as a loading control. The sizes of the protein standards are shown on the right side of the gel. The angled arrow indicates the position of the Strep-FeoB polypeptide. Extracts derived from MC4100 and PM06 transformed with pFEO did not synthesize Strep-tagged FeoB and therefore acted as a negative control. Click here for file
