# Supplementary figures and images for: Iron restriction induces preferential down-regulation of H2-consuming over H2-evolving reactions during fermentative growth of Escherichia coli
Source: BMC Microbiol. 2011 Aug 31;11:196. doi: 10.1186/1471-2180-11-196 (PMC3176205; doi:10.1186/1471-2180-11-196)

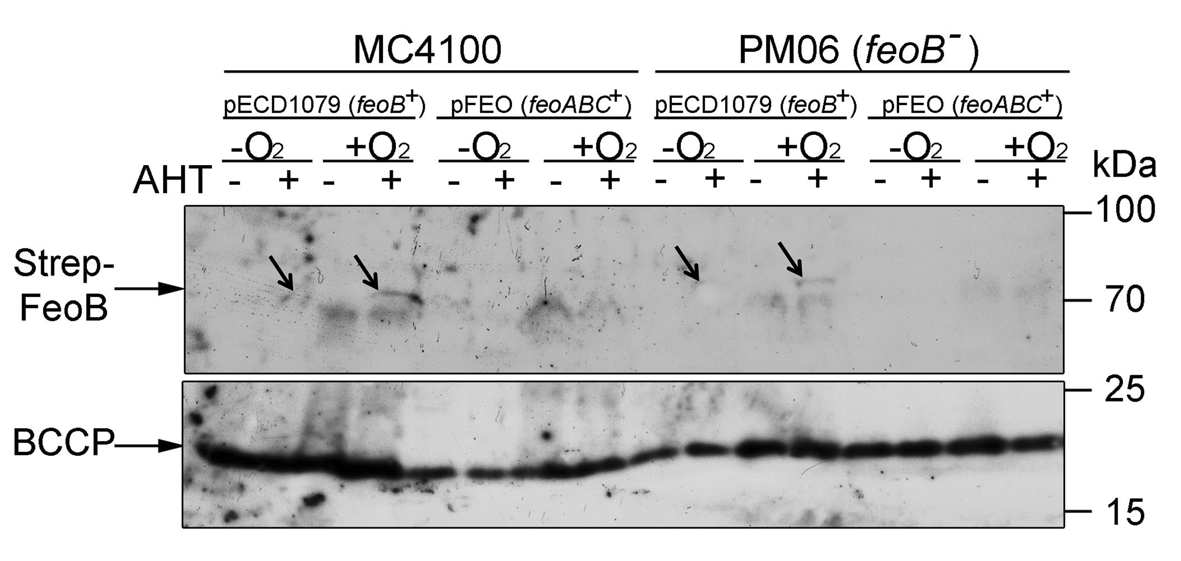

Supplement: Additional file 1 — Plasmid-encoded FeoB synthesis in MC4100 and PM06 (feoB::Tn5). Extracts (25 μg protein in membrane sample buffer) from MC4100 and PM06, transformed with pECD1079 bearing feoB and pFEO bearing the whole feo operon, both cloned behind a tetracycline promotor and encoding an N-terminal StrepII-tag on FeoB encoded on pECD1079 were separated by SDS-PAGE (10% w/v polyacrylamide) and after transfer to nitrocellulose detected by incubation with Strep-tactin conjugated to horseradish peroxidase. Strains were grown either with or without aeration in TGYEP, pH 6.5 and gene expression was induced with 0.2 μg ml-1 AHT (anhydrotetracycline) as indicated. Biotin carboxyl carrier protein (BCCP) served as a loading control. The sizes of the protein standards are shown on the right side of the gel. The angled arrow indicates the position of the Strep-FeoB polypeptide. Extracts derived from MC4100 and PM06 transformed with pFEO did not synthesize Strep-tagged FeoB and therefore acted as a negative control. [file 1471-2180-11-196-S1.TIFF]
